# Chronic pain and premature mortality in men and women, using data from UK Biobank

**DOI:** 10.1172/JCI166949

**Published:** 2023-03-01

**Authors:** Gary J. Macfarlane, Marcus Beasley, Gareth T. Jones

**Affiliations:** Aberdeen Centre for Arthritis and Musculoskeletal Health (Epidemiology Group), University of Aberdeen, Aberdeen, United Kingdom.

**Keywords:** Therapeutics, Pain

## To the Editor:

Muralidharan et al. (1) demonstrated that 4 months after nerve injury, male (but not female) mice demonstrated telomere length reduction and p53-mediated cellular senescence in the spinal cord, which was associated with pain chronicity and a decreased lifespan. They analyzed data from UK Biobank and concluded that their sex-specific observations in mice of a decreased lifespan were replicated in humans. The basis of this conclusion was a finding that among men in UK Biobank, a larger number of pain sites reported at recruitment associated with an earlier age at death (slope –0.19, *P* = 0.00011), while among women, the relationship between pain sites and death was much weaker and not statistically significant (slope –0.039, *P* = 0.51). We believe that the method of analysis was inappropriate, and the authors have come to an incorrect conclusion on this specific point.

If one wishes to examine the risk associated with pain status and death, the optimal approach is to follow participants prospectively and to quantify the risk of death in relevant groups. Muralidharan instead only considered participants who had already died and ignored data from people who were yet to die, despite the fact that they also contribute informative data to the question being answered.

We have previously published UK Biobank data examining the relationship between chronic widespread pain (CWP) and premature mortality (2). In brief, we identified persons who reported CWP at the time of recruitment, 2006–2010, and prospectively identified deaths up until August 2015. Persons with CWP were more than twice as likely to die in the follow-up period (mortality risk ratio [MRR], 2.43; 95% CI, 2.17, 2.72). We did not, however, examine whether the effect on mortality was different between men and women. We have therefore rerun our previous analysis (with data on death until April 2022; see Table 1). The MRRs were almost identical in men and women (MRR, 2.28 [95% CI, 2.10, 2.48] and MRR, 2.31 [95% CI, 2.12, 2.52], respectively). We had previously shown that the effects of CWP on excess mortality were mediated primarily through high BMI, low levels of physical activity, and other lifestyle factors, such as smoking, alcohol drinking, and markers of healthy diet. After adjustment for these (and age) in sex-specific models, the results were still very similar (MRR, 1.38 in men and 1.47 in women). In a single fully adjusted model involving both sexes, the MRR associated with CWP was 1.47 (95% CI, 1.30, 1.67), that for male sex was 1.63 (95% CI, 1.57, 1.70), and the interaction term for having CWP and being male was 0.94 (95% 0.79, 1.11). Mhuralidharan et al. (1) instead examined the data on mortality risk by number of pain sites reported. We therefore reanalyzed the data using number of pain sites in the fully adjusted model. Risk of death increased with number of pain sites reported. The interaction terms between number of sites and sex were all close to 1 (ranging between 0.92 and 1.10), and none were statistically significant.

Not only do the data not support the hypothesis that the relationship between CWP and excess mortality is specific to male sex, but they provide strong evidence that the association exists in both sexes and is of near-identical magnitude. This indeed is not surprising, given that we have shown that the effects of pain on mortality are importantly mediated through low levels of physical activity and high BMI, and these are likely sequelae in both sexes. Our analysis of the data from UK Biobank does not of course invalidate the mouse and genetic data reported in Muralidharan et al. (1), but it is important to set on record that the sex-specific effect of pain on longevity is not observed in humans.

## Figures and Tables

**Table 1:**
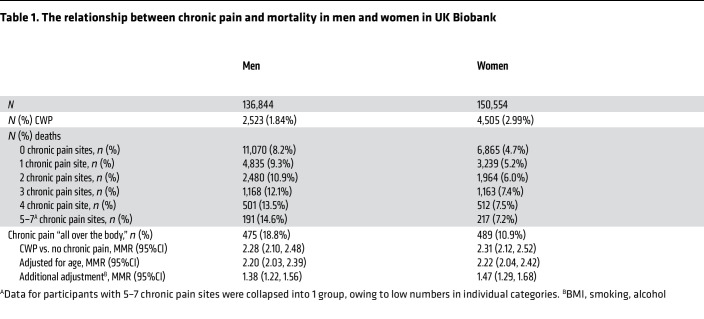
The relationship between chronic pain and mortality in men and women in UK Biobank

## Version 1 03/01/2023

Electronic publication
